# Preparation of thiourea derivative incorporated Ag_3_PO_4_ core shell for enhancement of photocatalytic degradation performance of organic dye under visible radiation light

**DOI:** 10.1038/s41598-024-62608-9

**Published:** 2024-06-03

**Authors:** Omnia A. A. El-Shamy, Hanaa Selim, Ahmed S. Elkholy, Rasha S. Kamal, Nashwa M. Saleh, Nour E. A. Abd El-Sattar

**Affiliations:** 1 Department of Analysis and Evaluation, Egyptian Petroleum Research Institute, Nasr City, Cairo, 11727 Egypt; 2 Main Defense Chemical Laboratory (M.D.C.L.), Almaza, Cairo, Egypt; 3 Department of Petroleum Applications, Egyptian Petroleum Research Institute, P.O. Box 11727, Nasr City, Cairo, Egypt; 4 Department of Chemistry, Faculty of Science (Girls), Al-Azhar University, Yousef Abbas Str., P.O. Box: 11754, Nasr City, Cairo, Egypt; 5 Department of Chemistry, Faculty of Science, Ain Shams University, Cairo, 11566 Egypt; 6 Basic & Medical Sciences Department, Faculty of Dentistry, Alryada University for Science & Technology, Sadat City, Egypt

**Keywords:** Silver phosphate, Photocatalytic activity, Photoluminescence, Band gap, Thermal stability, Environmental impact, Sustainability

## Abstract

Photocatalysis is a promising technique to reduce hazardous organic pollutants using semiconductors under visible light. However, previous studies have been concerned with the behavior of silver phosphate (Ag_3_PO_4_) as n-type semiconductors, and the problem of their instability is still under investigation. Herein, 4,4′-(((oxalylbis(azanediyl)) bis(carbonothioyl)) bis(azanediyl)) dibenzoic acid is synthesized by green method and used to enhance the photocatalytic behavior for Ag_3_PO_4_. The incorporated Ag_3_PO_4_ core–shell is prepared and characterized via XRD, FT-IR, Raman, TEM and BET. Besides, the thermal stability of the prepared core shell was investigated via TGA and DSC measurements. The optical properties and the energy band gap are determined using photoluminescence and DRS measurements. The photodegradation of methylene blue in the presence of the synthesized Ag_3_PO_4_ core–shell under visible light is examined using UV/Vis measurements. The effect of initial dye concentration and contact time are studied. In addition, the kinetic behavior of the selected dye during the photodegradation process shows a pseudo-first order reaction with rate constant of 0.015 min^−1^ for ZAg. The reusability of the Ag_3_PO_4_ core shell is evaluated, and the efficiency changed from 96.76 to 94.02% after three cycles, indicating efficient photocatalytic behavior with excellent stability.

## Introduction

The leading cause of environmental pollution, which harms both people and aquatic life in the water ecosystem, is the release of highly toxic inorganic and organic pollutants from various industries to the water surface^1–3^. Energy and the environment are two of the biggest problems associated with the growth of humanity, the advancement of industrialization, and the vast usage of fossil fuels^4,5^. The majority of conventional pollution control methods have issues like high costs and lengthy implementation times. They are challenging to fulfill environmental management demands^2,6^.

Photocatalysis prevents secondary pollutants, saves energy, and operates efficiently^7,8^. There are numerous uses for photo-catalysis in the cleanup of the environment^9–11^. Traditional photo-catalysts like ZnO and TiO_2_ have a lower quantum efficiency and can only absorb UV light^12,13^. They need to meet the requirements of extensive practical applications. Therefore, it is essential to discover a visible light photo-catalyst that is more effective and reliable.

With plenty of solar light available, semiconductor photocatalysis has been viewed as a potentially promising method for addressing current energy and environmental issues^14–16^. However, the photocatalytic efficiency of the available semiconductor materials are typically weak in the visible light range or has insufficient charge separation ability^17,18^. In order to solve this problem, much work has been done in recent years to discover and create novel semiconductor catalysts with enhanced photocatalytic capabilities^19,20^. Ag_3_PO_4_ is a brand-new n-type semiconductor that has an indirect bandgap width of 2.45 eV and can absorb both ultraviolet and visible light. Ag_3_PO_4_ has lower edge potentials than ZnO, with the conduction band edge potential being 0.4 eV and the valence band edge potential being 2.9 eV, respectively^21^. The photo-generated holes provide strong oxidizing power due to the exceptionally low valence band potential. Ag_3_PO_4_ has remarkable photo-oxidation capabilities because of its band gap and band potential, including the oxidation of organic dyes under visible light and the creation of oxygen^22,23^.

Hybrid photocatalyst is recommended and concerned by different research^24,25^. In 2023, Ansari^26^ Compared the photocatalytic performance of red phosphorus/ZnO nanohybrids by the non-hybrid catalyst. The results showed superior visible-light-driven photocatalytic activity toward the destruction of methyl orange dye, further demonstrating the potential of the ideal reaction parameter to increase visible light photocatalytic activity effectively.

Ag_3_PO_4_ has numerous benefits, including excellent efficiency of both photocatalytic behavior and quantum especially when at irradiation wavelength of > 420 nm^23,27^, its insolubility in water, and a better conduction band.

The primary purpose of Ag_3_PO_4_ and other semiconductors is to guide the transmission of the built-in electric field-driving carrier, which allows the separation of electron/hole (e/h)^28^. This lessens Ag_3_PO_4_ corrosion and enhances photocatalytic activity^29–31^. Ag_3_PO_4_'s e- on the conduction band are either transported to other semiconductors or are abundant in free radical linking reactions (for example, the reduction of oxygen) that decrease the silver ion liberation from the Ag_3_PO_4_ lattice. Because Silver ions are vulnerable to photo-corrosion to create Ag, Ag_3_PO_4_ improvement is recommended for use as a photocatalyst. In order to increase Ag_3_PO_4_'s photocatalytic activity, researchers have conducted in-depth studies on the modification of this compound using various techniques. The use of the organic compounds as an outer coating for the Ag_3_PO_4_ supports the electronic activity and delays their combination. In addition, heterorganic compound possesses additional properties such as corrosion resistance by donating their electron^32–34^.

In this work, the composite structure, the core–shell structure, is used to improve the photocatalytic activity of Ag_3_PO_4_. The green synthesis method is used to prepare 4,4′-(((oxalylbis(azanediyl))bis(carbonothioyl))bis(azanediyl))dibenzoic acid abbreviated as Z. The prepared Z is characterized via FT-IR, then it used to prepare the incorporated Ag_3_PO_4_ core–shell as an enhanced photocatalytic material abbreviated as ZAg. The synthesized photocatalyst is characterized using FT-IR, XRD, Raman, BET and TEM techniques and its band gap is calculated from DRS measurements. The thermal stability is determined using TGA and DSC measurements. The efficiency of ZAg as photocatalytic material on Methylene blue (MB) was investigated under the irradiation of visible light using UV/Vis measurements. The stability and the reusability of the synthesized Zag were also examined.

## Experimental sections

### Chemicals and materials

Silver nitrate, Ammonium dihydrogen phosphate, Oxalyl chloride, and antharanilic acid, were obtained from the American company Sigma-Aldrich in Egypt, NaOH powder was obtained. Methylene blue dye obtain from Merck. Freshly deionized water was used to prepare the dye solutions for all products.

### Synthesis of 4,4'-(((oxalylbis(azanediyl))bis(carbonothioyl))bis(azanediyl)) dibenzoic acid

**Scheme 1:**
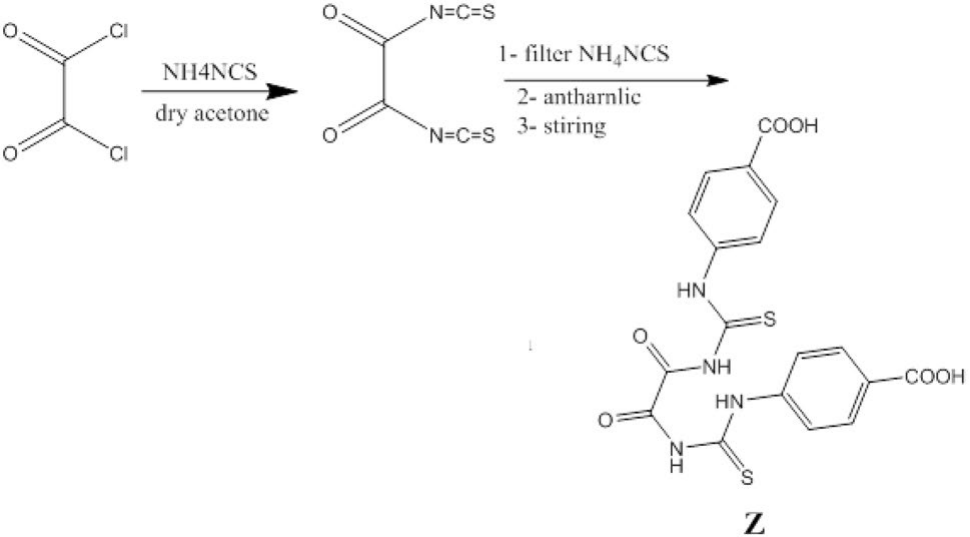
Synthesis of 4,4′-(((oxalylbis(azanediyl))bis(carbonothioyl))bis(azanediyl)) dibenzoic acid.

Stirring oxlayal chloride (1.27 gm, 100 mmoles) with ammonium thisyanate (1.52 gm, 200 mmoles) in 50 ml dry acetone for 10 min, then filter the product to remove ammonium chloride after that we add antharnilic acid ((2.74 gm, 200 mol) with stirring for 20 min, furthermore, we evaporate the acetone to abstain the product, then we crystalize it from ethanol to obtain yellow crystal. Scheme 1 show the preparation of 4,4′-(((oxalylbis(azanediyl))bis(carbonothioyl))bis(azanediyl)) dibenzoic acid.

### Synthesis of Ag_3_PO_4_ nanoparticles

Green synthesis methods are used for the preparation without the addition of any solvents^35–37^. The co-precipitation was applied to synthesize the Ag_3_PO_4_ nanoparticles, and the procedure was as follows: a suitable quantity of AgNO_3_ salt was completely dissolved in 50 ml of deionized water while continuously stirring. Also, an equivalent volume of crystalline NH_4_H_2_PO_4_ was dissolved in 50 ml of deionized water with constant magnetic stirring. The second solution, comprising NH_4_H_2_PO_4_, was then gradually added to the first solution, containing AgNO_3_, while being continuously stirred until forming a yellow precipitate. The precipitate was then crushed and calcined at 400 °C for one hour to produce the Ag_3_PO_4_ nanocrystalline sample, which was then ready for usage.

### Synthesis of core–shell

**Scheme 2:**
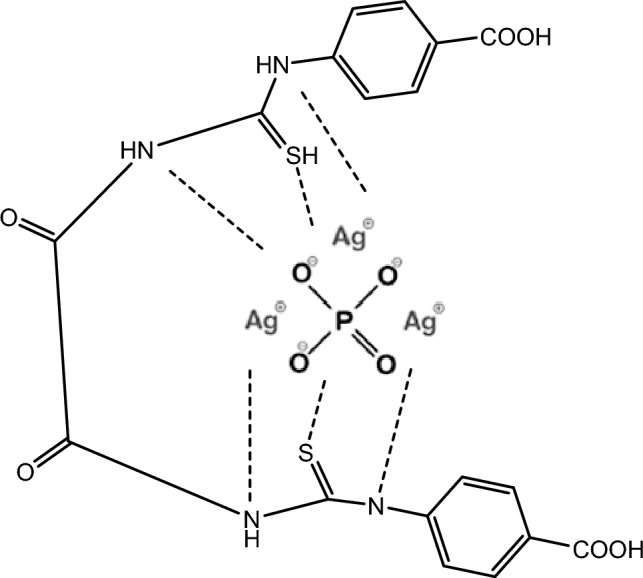
Schematic representation of the synthesized ZAg.

A calculated amount of the Z as shell was combined with the silver phosphate core, followed by a 10-min period of rigorous grinding to produce a homogeneous coating of the Z over the Ag_3_PO_4_ core. To produce prepared core–shell nanoparticles, the resultant mixture was heated at a 3°/min for 1 h in a muffle furnace at 400 °C. Scheme 2 declares the structure of the synthesized compound.

### Characterization techniques

Using the Philips X'Pert X-ray diffraction (XRD) system with Cu–Ka radiation (= 1.54056 Å), the crystallographic pattern of the produced nanocomposite was examined. A typical 2 scan had a scan speed of 4o/min and ranged from 10° to 80°. The functional groups were identified using Nicolet iS10 FT-IR spectroscopy, which uses Fourier-transform infrared spectroscopy. A JEOL-JEM 3200 electron microscope operating at 300 kV was used to capture TEM pictures. To examine the optical characteristics of the produced materials, a Shimadzu IRS-2200 diffuse reflection (DRS) system in combination with an ultraviolet–visible spectrometer (Jasco V-507) was utilized. A Perkin Elmer fluorometer (model LS-55) was used to analyze solid-state photoluminescence (PL) at ambient temperature.

### Photocatalytic activity study

**Figure 1:**
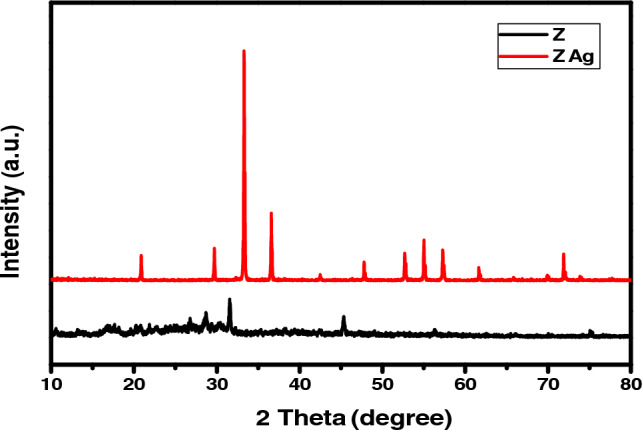
XRD spectra of the synthesized Z and ZAg.

500 Watt halogen lamp used as a radiation source to evaluate the photocatalytic activity of ZAg for the degradation of the molecules of MB dye as a model of organic pollutants (as shown in Fig. 1S). In each test, 50 mL of the dye solution with varying initial concentrations of 10, 20, 35 and 50 ppm were taken in a glass beaker, and the effect of the prepared catalyst with 1.0 g/L were added. For 30 min, the mixture was agitated in the dark to achieve adsorption/desorption equilibrium between the catalyst and the MB dye molecule and then continued while being exposed to visible light while being constantly stirred. Then, 3 mL of suspensions were withdrawn regularly from the reactor at predetermined intervals (30, 60, 90, 120,150,180 and 210 min). ZAg was separated using a centrifuge, and the supernatant was measured using UV/Vis measurements at maximum absorption wavelength of 660 nm. Before the addition of ZAg, and after doing a blank experiment, it was discovered that dye degradation was constrained.

## Result and discussion

### Characterization of the synthesized materials

**Figure 2:**
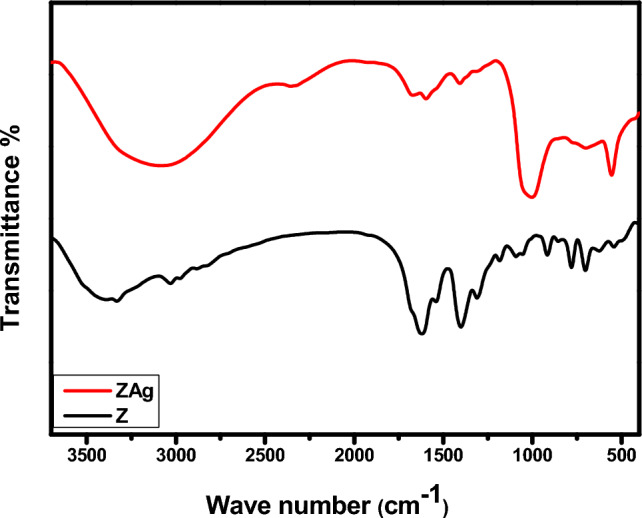
FT-IR spectra of the synthesized Z and ZAg core–shell.

The crystalinity of ZAg are confirmed with the sharp characteristic peaks illustrated in Fig. 1. Characteristic diffraction peaks are located at different 2θ (degrees) that are assigned to the indexed planes of silver phosphate that match with the previous research and the standard XRD data for Ag_3_PO_4_ JCPDS file NO. 00-006-0505 (see Fig. 2S). The peaks at 2θ and their related crystal plane is summarized as: 20.88° [1 1 0], 29.69° [2 0 0], 33.28° [2 1 0], 36.58° [2 1 1], 42.52° [2 2 0], 47.79° [3 1 0], 52.68° [2 2 2], 55.01° [3 2 0], 57.27° [3 2 1], 61.65° [4 0 0], 65.87° [4 1 1], 69.95° [4 2 0], 71.89° [4 2 1] and 73.96° [3 3 1]^38,39^. The average particle size of ZAg calculated using Debye–Scherrer equation^40,41^ and found to be 36.55 nm.

**Figure 3:**
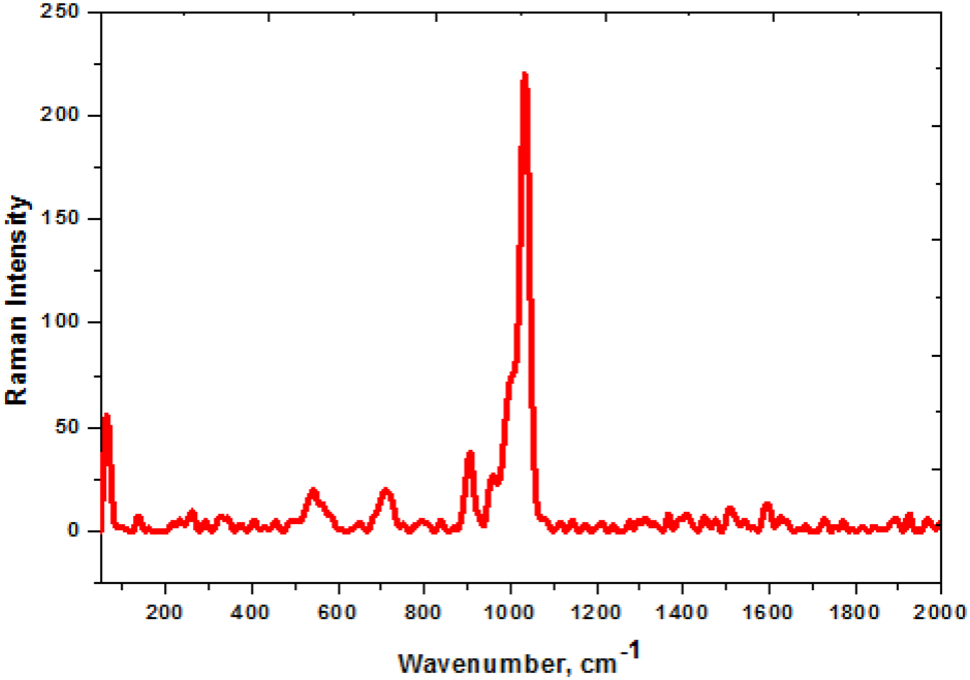
Raman spectra of the synthesized ZAg.

Figure 2 shows FT-IR spectra of Z and ZAg core–shell. Concerning the spectra of Z, the absorption band that present at 333.14 cm^−1^ corresponds to the stretching vibration of NH and OH groups^42,43^. Symmetric and asymmetric stretching vibration COO groups are located at 1400 cm^−1^ and 1620 cm^−1^, respectively^44,45^. The bands present at 1091.92 cm^−1^ and 1629.12 cm^−1^ are due to the stretching vibration of COH and C=O groups^46,47^. In addition, the absorption peak of S=O is located at 1184.09 cm^−1^, while that present at 1311.12 cm^−1^ is related to C–O and C–N groups. The peak found at 3030.64 cm^−1^ is due to the stretching vibration of the C–H group of the benzene ring^48,49^. The FT-IR spectra of ZAg show the characteristic peaks related to Z with low intensity confirming their interaction. Moreover, the peaks at 555.66 cm^−1^ and 1007 cm^−1^ are assigned to the bending vibration of O=P–O and asymmetric stretching vibration of O–P–O, respectively^50,51^. Besides, new peak present at 495 cm^−1^ in ZAg is attributed to the formation of Ag–N bond^40^. The previous data confirm not only the formation of Z (due to the presence of all the prepared functional groups) but also the formation of ZAg (see Fig. 3).

Raman spectroscopy is applied as a complement analysis to FT-IR is measured for the ZAg and displayed in Fig. 3. The symmetrical vibration of the AgO bending bond is located at 113 cm^−1^^52^. The peak located at 500 cm^−1^ are attributed to the symmetrical str. vibration of P–O–P bonds^53^. In addition, the peak present at 919.13 cm^−1^ is due to the vibration of the terminal oxygen of the phosphate group^51,54^. The aromatic ring and C=S are strong peaks in the range 995–1050 cm^−1^^55,56^.

**Figure 4:**
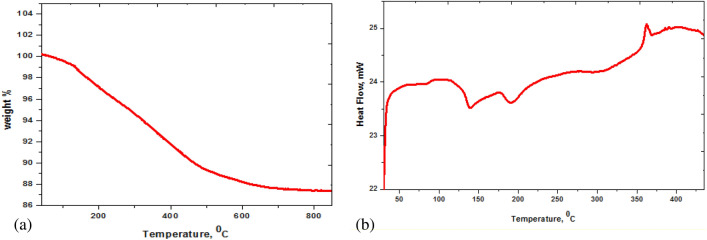
(**a**) TGA curve, and (**b**) DSC curve of ZAg core–shell.

The stability of the photocatalysts is an important parameter that determines their efficiencies over higher temperatures^57^. Figure 4a declares the thermal stability of ZAg using Thermal gravimetric analysis. The decomposition of any impurities and the adsorbed water molecules on the surface of the investigated photocatalytic substances (ZAg) is present in the 100 to 200 °C range. Then, the decomposition of organic material started until it reached complete decomposition around 550 °C. Excellent thermal (> 800 °C) stability of the Ag_3_PO_4_ was observed and matched with the literature^58^.

Besides, the DSC of ZAg is determined and displayed in Fig. 4b. A weight loss of ≈10% accompanies the sharp endo peak near 400 °C (see Fig. 4), confirming the starting decomposition of the organic molecules (Z).

**Figure 5:**
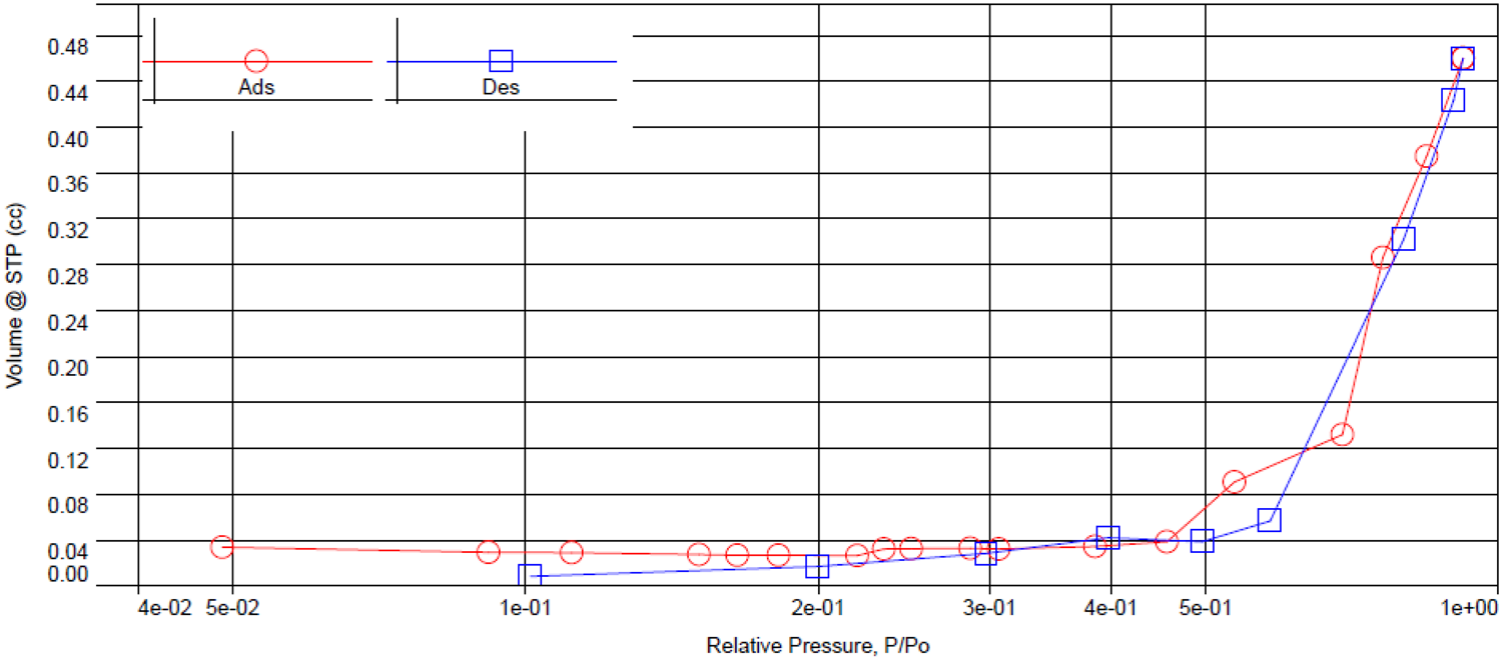
The adsorption–desorption isotherm of ZAg.

The surface area of the investigated photocatalyst was determined via BET measurements and found to be 0.395 m^2^/g, indicating moderate surface area. Concerning Fig. 5, The synthesized ZAg shows Type IV isotherm with H3 hysteresis determined from Nitrogen adsorption–desorption isotherm. The previous results suggest that ZAg is a mesoporous material^59^.

**Figure 6:**
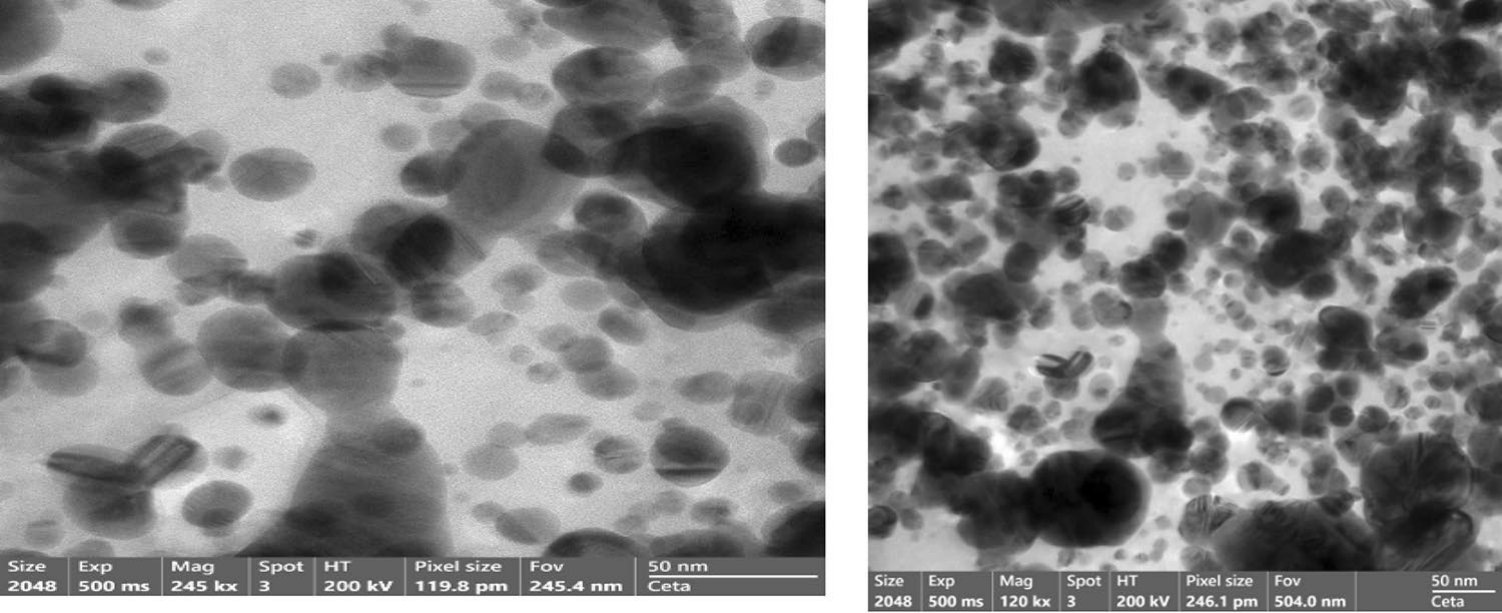
TEM image of ZAg with different scale.

The morphology of the synthesized ZAg was obtained from TEM images (see Fig. 6) Irregular spherical particles of ZAg are distributed indicating the coating of the Ag_3_PO_4_ with Synthesis of 4,4′-(((oxalylbis(azanediyl))bis(carbonothioyl))bis(azanediyl)) dibenzoic acid. Besides, the image shows aggregation of the core shell Ag_3_PO_4_ that occur due to the intermolecular interaction between the coated Z. the previous data is matched with FT-IR and confirming the formation of heterojunction photocatalyst.

**Figure 7:**
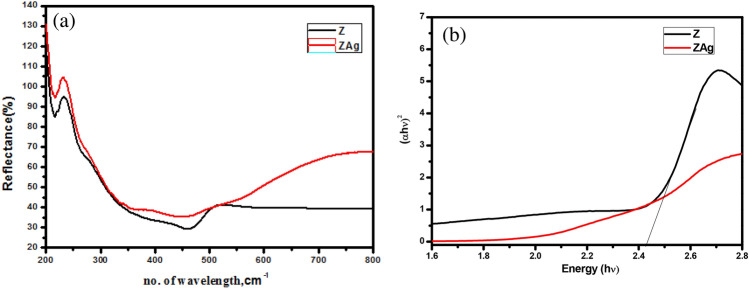
(**a**) UV/Vis diffuse reflectance spectra and (**b**) Band gap spectra of ZAg core–shell.

Using UV/Vis—DRS optical spectroscopy in the 200–800 nm region, the diffuse reflection spectra of the Z and ZAg core–shell were examined (see Fig. 7a). It has been found that the ZAg core–shell absorbs visible light at a substantially faster rate than the Z.

The production of electron–hole pairs, light absorption, charge carrier transfer, and charge carrier utilization are a few of the mechanisms that control photocatalytic activity^60^. The photocatalytic material's energy band gap (E_g_), which affects the efficiency of the product and the transfer of the e/h pairs, is crucial for maximizing the photocatalytic activity. The graph relationship of the Kubelka–Munk equation was used to determine the E_g_ value^61,62^:

1 $$\alpha hv = (A\left( {hv - E_{g} } \right))^{\frac{n}{2}}$$

where n: the proportionality constant (n = 1), v: the frequency of light, and α: the absorption coefficient.

**Figure 8:**
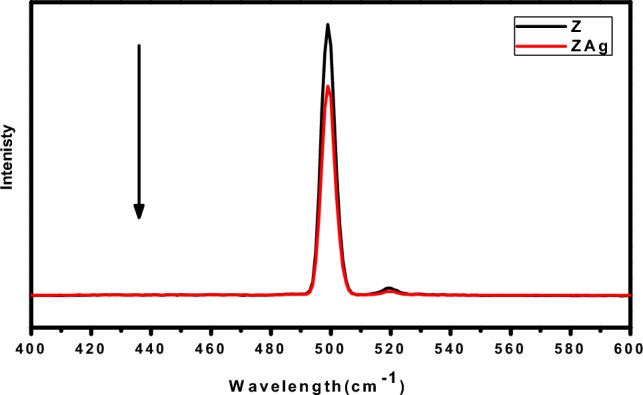
Photoluminescence spectra of pure Z and ZAg.

The diffuse reflectance spectra and the Z and ZAg band gab are illustrated (see Fig. 8a and b). As shown in Fig. 7b, the optical band gaps were computed using plots of (αhʋ)^2^ versus photon energy (hʋ). The band gap narrowed due to the conjugation of two bands, increasing the stability of the e/h couples (quantum confinement effect). The ZAg core shell has a band gap of 2.24 eV which is lower value than the value of band gap for pure Ag_3_PO_4_ (2.39 eV)^63^ indicating that the formed core shell silver phosphate (ZAg) move towards the redshift.

Mulliken electrochemical equation are used to determine the energy of valence band (E_VB_) and the energy of the conduction band (E_CB_)^64^:

2 $$E_{VB} = x - E_{e} + 0.5E_{g}$$

3 $$E_{CB} = x - E_{e} - 0.5E_{g}$$

where, ${E}_{e}$: the energy of free electron (4.5 eV), ${E}_{g}$: the energy of band gap, and $x$: the electronegativity of Ag_3_PO_4_ = 5.96 eV^63^. The estimated ${E}_{CB}$ and ${E}_{VB}$ of Ag_3_PO_4_ are found to be − 0.265 eV and 2.655 eV, respectively.

The PL spectra of pure Z and ZAg at a 370 nm excitation wavelength are shown in Fig. 8. The PL spectra show the rate of charge separation and recombination within the photocatalysts. The rate of charge separation and recombination within the photocatalysts is shown by the PL spectra. As a result of the effective charge transfer at the heterostructure interface, the PL spectrum of pure Z has a prominent emission band at 498 nm. At the same time, the PL intensity of the ZAg core–shell has significantly reduced, indicating that the recombination between photo-electrons generated and holes has decreased. Additionally, pure Z and ZAg as a heterostructure have a synergistic effect of reducing PL intensity, which increases the lifetime of electron stability^65,66^.

### Photocatalytic activity of ZAg Core–shell

#### Effect of the intial dye concentration

**Figure 9:**
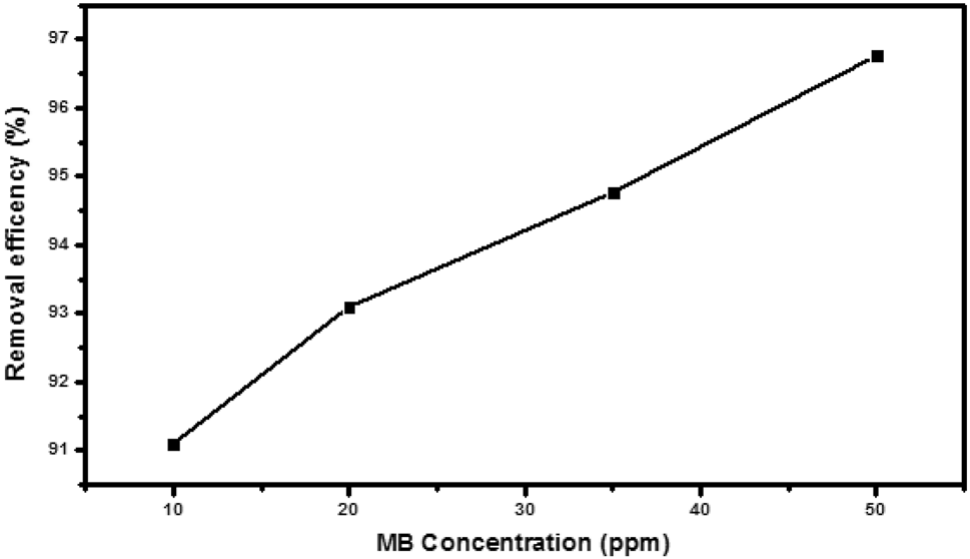
The effect of MB degradation using ZAg after 120 min of irradiation.

According to Fig. 9, which reveals that the best effectiveness was seen at an MB starting concentration of 50 ppm, the influence of MB initial concentration was examined depending on UV/Vis measurements in the range of 10–50 ppm.

It is clear that the removal efficiency increase significantly with the initial concentration of dye from 1–20 ppm. In the range from 20–40 ppm and 40–50 ppm the MB degradation increase slowly (around 2%) with increasing the initial dye concentration due to the trapping of photon to the solution.

#### Effect of contact time

Figure 8a depicts the photocatalytic degradation of MB dye under irradiation of visible light in the presence of Z and ZAg core shells with time irradiation of 0, 30, 60, 90, 120, 150, 180, and 210 min. It is evident that the ZAg is more photocatalytically active than Z for the MB degradation (see Fig. 11a). The photocatalyst's MB solution was left in the dark for 30 min before initiating the photocatalytic process to achieve the adsorption–desorption equilibrium (see Fig. 3S). The relation between the dye concentration in the absence and in the presence of ZAg is illustrated in Fig. 4S.

**Figure 10:**
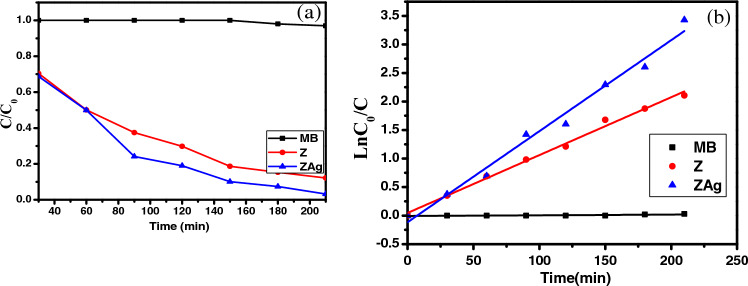
(**a**) The photocatalysis degradation curve of Z and ZAg under irradiated light (after 30 min dark), (**b**) Kinetic behavior of MB degradation using Z and ZAg under irradiation of visible light.

The following relation provided the efficiency of the deterioration of MB under visible light:

4 $${\text{D}}\% = \left( {\frac{{C_{0} - C}}{{C_{0} }}} \right)*100$$

where C_0_ and C: the initial and the remaining concentration of the investigated dye. As shown in Fig. 10a, the efficiency of Z and ZAg as photocatalytic martials was found to be 87.82% and 96.76%, respectively after 210 min.

The next pseudo-first-order equation represents the kinetics of the photocatalytic degradation reaction of MB in accordance with the L–H kinetics model.

5 $$\ln \left( {\frac{{C_{0} }}{C}} \right) = k_{a} *t$$

where k_a_: the rate constant (min^−1^), the concentration in mg L^−1^ and t: time in min. Concerning Fig. 10b, K_a_ is the slope of the linear relation between ln (C_0_/C) and t and found to be 1.240 × 10^–4^, 0.010, and 0.015 min^−1^ for MB, Z, and ZAg, respectively. The rate constants are increased in the following order: ZAg > Z > MB (see Fig. 5S), indicating excellent catalytic efficiency of the ZAg core–shell. From all the previous data, ZAg core–shell can work as an effective photocatalyst for organic compound degradation with good stability. The photocatalytic degradation of similar materials listed in Table 1.

**Table 1:** Photocatalytic activities of various photo-catalyst and organic pollutants.

Photocatalytic material	Organic pollutant	Time, min	Wavelength, nm	Lamp	Power, W	Efficiency, %	References
Ag_3_PO_4_–75% TiO_2_	MB	4	665	Tungsten Halogen lamp	300	97.1%	^67^
Ag_3_PO_4_–75% TiO_2_	pyrimethanil	6	270	Tungsten Halogen lamp	300	98%	^67^
Ag_3_PO_4_/GO	Methyl orange	50	420	Halogen lamp	500	86.4%	^68^
g-C3N4/Ag^3^PO_4_	Rhodamine B	10	420	Xenon lamp	300	96%	^69^
Bi_4_-xLaxTi_3_O_12_ 0 ≤ x ≤ 1	Rhodamine B	240	253.4	low-pressure lamps	35	99%	^70^
AgBr/Ag_3_PO_4_	Methyl orange	40	420	Xenon lamp	500	95.1%	^71^
Ag_3_PO_4_/AgBr	MB	21	400	Xenon lamp	150	96%	^72^
ZAg	MB	120	570	Halogen		96.76%	This work

### The photocatalytic mechanism

Reactive oxidizing species were produced concurrently with the photocatalytic process to accomplish the photocatalytic destruction of organic contaminants. Two different processes produce reactive oxidizing species: first, the adsorbed water is oxidized with the help of the photocreated holes (h^+^), and second, the photostimulated electrons reduce the oxygen that has been adsorbed on the surface. The excited electrons (e^−^) in the conduction band will interact with the surface-adsorbed oxygen to produce reactive superoxide anion radicals (^−^O_2_^·^) when the adsorbed OH on the photocatalyst interacts with the reactive holes in the valence band, resulting in the formation of hydroxyl radicals (OH^·^). The two most well-known and influential oxidizing species are OH^·^ and ^−^O_2_^·^^65^.

**Figure 11:**
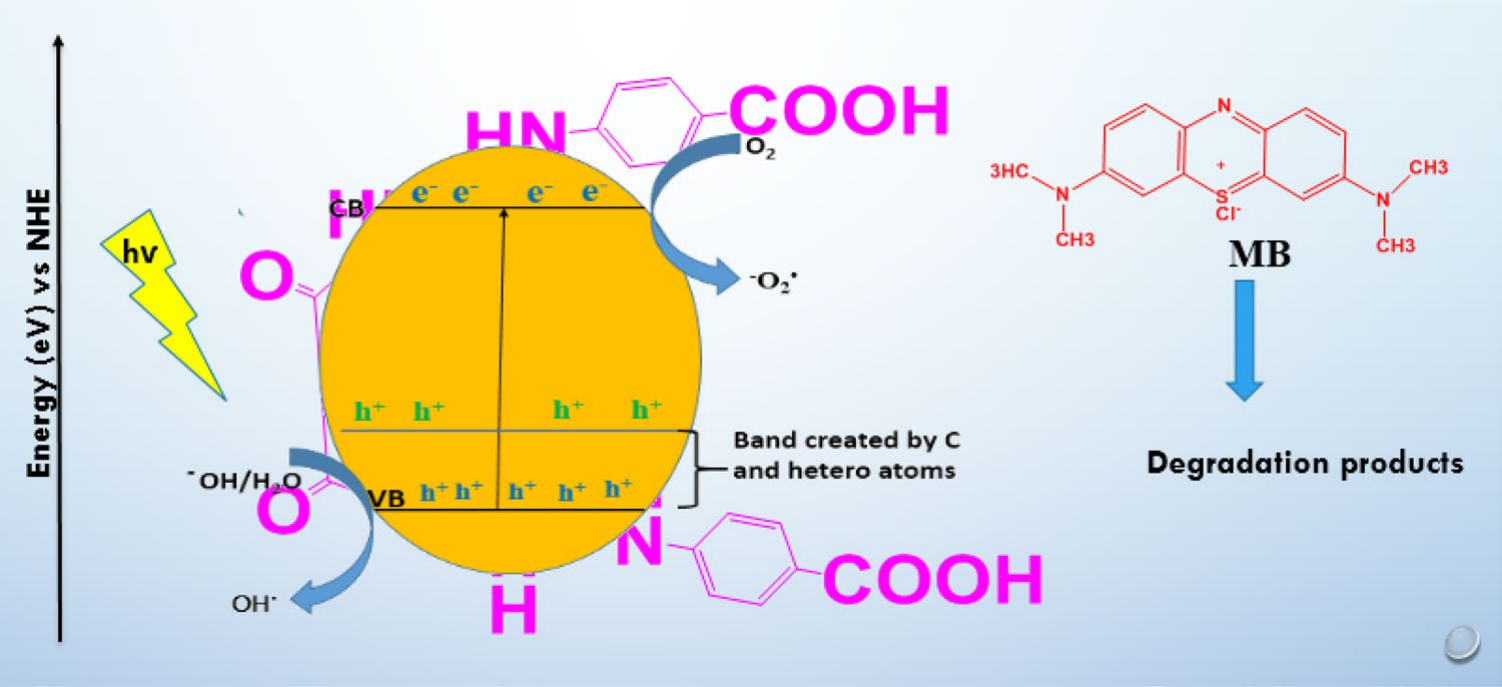
Schematic representation of the photo-degradation process.

In this instance, the two reactive species were involved in the photocatalytic degradation of MB. Organic molecules-derived carbon, sulfur and nitrogen decrease the Ag_3_PO_4_ band gap by generating energy levels above the Ag_3_PO_4_ valence band (VB) and oxygen vacancies. When visible light excites the electrons in the conduction band (CB) of Ag_3_PO_4_ band, these photostimulated electrons move from the (CB) to the lower CB. As a result, the transferred electrons' reduction potential will decrease. The potent reactive oxidizing species (OH^·^ and ^−^O_2_^·^) produced by these excited photocarriers will cause the photodegradation of MB and the conversion into H_2_O and CO_2_. The photodegradation mechanism is represented in Fig. 11.

### The reusability and stability of ZAg after photocatalytic degradation reaction

**Figure 12:**
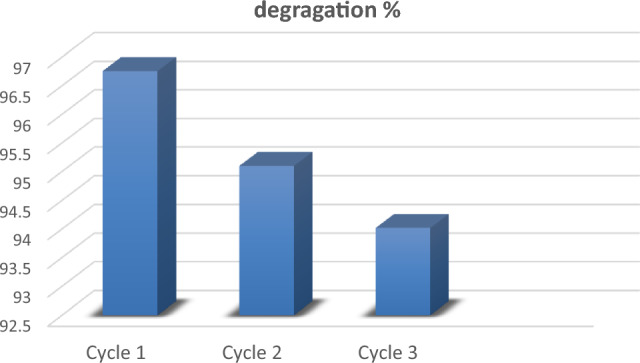
The degradation percentage of MB after three cycle using ZAg.

The effectiveness of the photocatalytic degradation of MB using ZAg for three cycles under visible light illumination was 94.02% (See Fig. 12).

**Figure 13:**
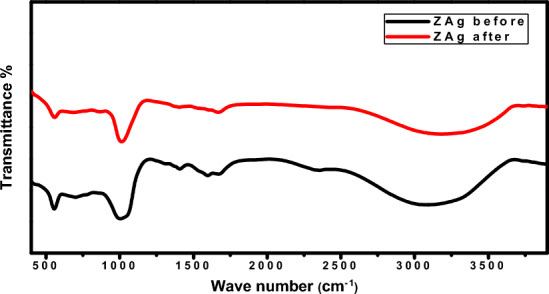
FT-IR spectra of ZAg bfeore and after photocatalytic degradation of MB under visible light.

The FT-IR measurements (see Fig. 13) utilized to verify the stability of the synthesized materials after three cycles further show that ZAg has been applied as easily manufactured with low environmental impact and considered efficient and economical photocatalysis for organic pollutants under visible light.

## Conclusion

Photodegradation enhancement of organic pollutants in aqueous media under visible light using photocatalysis consider serious challenge. Therefore, Ag_3_PO_4_ is prepared and enhanced by incorporating with 4,4′-(((oxalylbis(azanediyl))bis(carbonothioyl))bis(azanediyl)) dibenzoic acid forming ZAg. The performance of ZAg as photocatalytic material on MB under visible light was evaluated using UV–Vis measurements and found to be 96.76%. Excellent stability of the investigated photocatalytic materials up to 800 °C was confirmed via DCS and TGA measurements. The effect of initial concentration of the investigating dye and the contact time was determined. The photodegradation reaction of MD under visible light in the presence of ZAg is pseudo-first order. The data show that ZAg is highly efficient and stable after three cycles.

### Supplementary Information

Supplementary Figures.

## Data Availability

The datasets used and/or analyzed during the current study available from the corresponding author on reasonable request.
