# Supplementary material for: Preparation of thiourea derivative incorporated Ag3PO4 core shell for enhancement of photocatalytic degradation performance of organic dye under visible radiation light
Source: Sci Rep. 2024 Jun 3;14:12671. doi: 10.1038/s41598-024-62608-9 (PMC11148051; doi:10.1038/s41598-024-62608-9)
Supplement: Supplementary file 1 — Supplementary Figures. [file 41598_2024_62608_MOESM1_ESM.docx]

**Preparation of thiourea derivative incorporated Ag_3_PO_4_ core shell for enhancement of photocatalytic degradation performance of organic dye under visible radiation light**

**Omnia A. A. El-Shamy^1,*^, Hanaa Selim^1^, Ahmed S. Elkholy^2^, Rasha S. Kamal^3^, Nashwa M. Saleh^4^, Nour E. A. Abd El-Sattar^5,6^.**

***Corresponding author:** [**omniaelshamy@yahoo.com**](mailto:omniaelshamy@yahoo.com)

**^1^ *Department of Analysis and Evaluation, Egyptian Petroleum Research Institute, Nasr City, Cairo, 11727, Egypt.***

***^2^ Main Defense Chemical Laboratory (M.D.C.L), Almaza, Cairo, Egypt***

***^3^ Department of Petroleum Applications, Egyptian Petroleum Research Institute, Nasr City, P.O. Box 11727, Cairo, Egypt.***

***^4^ Department of Chemistry, Faculty of Science (Girls), Al-Azhar University, P.O. Box: 11754, Yousef Abbas Str., Nasr City, Cairo, Egypt***

***^5^ Department of Chemistry, Faculty of Science, Ain Shams University, Cairo, 11566, Egypt.***

***^6^ Basic & Medical Sciences Department, Faculty of Dentistry, Alryada University for Science & Technology. Egypt***


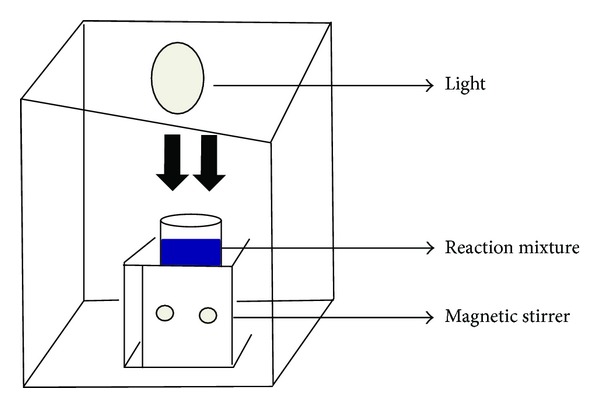


**Fig. 1S.** Schematic diagram of the photocatalytic experimental set-up.


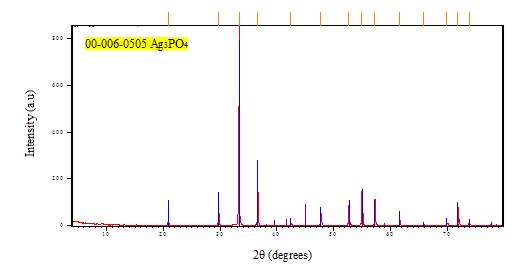


**Fig. 2S**: The matched XRD spectra of ZAg with Ag_3_O_4_

(JCPDS file no. 00-006-0505)


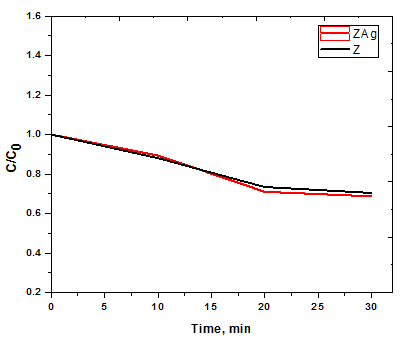


Fig. 3S: The photocatalysis degradation curve of Z and ZAg under dark condition.


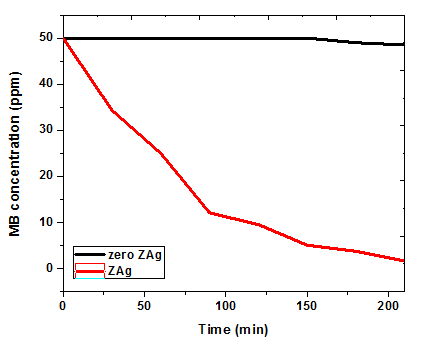


Fig. 4S: The decrease in the concentration of MB in the absence and presence of photocatalyst.

Fig. 5S: The photocatalysis degradation curve of Z and ZAg under dark condition.
